# Protective Effects of Shenfuyixin Granule on H_2_O_2_-Induced Apoptosis in Neonatal Rat Cardiomyocytes

**DOI:** 10.1155/2021/6654457

**Published:** 2021-01-29

**Authors:** Xinlu Wang, Xuanxuan Hao, Youping Wang, Bin Li, Lin Cui, Shiyang Xie, Yongxia Wang, Mingjun Zhu

**Affiliations:** ^1^The First Affiliated Hospital of Henan University of CM, Zhengzhou, Henan, China; ^2^Henan University of Chinese Medicine, Zhengzhou, Henan, China

## Abstract

Shenfuyixin granule (SFYXG, i.e., Xinshuaikang granule) is a prescription, commonly used in the clinical experience, which plays a significant role in the treatment of heart failure. The purpose of this present research was to investigate the protective effect of SFYXG, and the mechanism about anti-H_2_O_2_-induced oxidative stress and apoptosis in the neonatal rat cardiomyocytes. Myocardial cells, as is well known, were divided into 4 groups: normal, model, SFYXG, and coenzyme Q10 group, respectively. Cells viability was determined by MTT assay. Flow cytometry and AO/EB staining were implemented to test the apoptosis rate and intracellular reactive oxygen species (ROS) level. Mitochondrion membrane potential (MMP) was evaluated by JC-1 fluorescence probe method. The myocardial ultrastructure of mitochondrion was measured by electron microscope. The related mRNA expression levels of Bax, Bcl-2, Caspase-3, caspase-8, and caspase-9 were detected by real-time polymerase chain reaction (PCR). Also, the expression levels of Bax and Bcl-2 protein were detected by Western blot, and the expression levels of caspase-3, caspase-8, and caspase-9 protein were tested by caspase-Glo®3 Assay, caspase-Glo®8 Assay, and caspase-Glo®9 Assay, respectively. GAPDH was used as the internal reference gene/protein. The results revealed that SFYXG (0.5 mg/ml) raised the viability of myocardial cell, weakened the apoptosis rate and ROS level, corrected the mitochondrion membrane potential stability, and improved cell morphology and ultrastructure of myocardial mitochondrion. Furthermore, SFYXG upregulated the antiapoptosis gene of Bcl-2, but downregulated the proapoptosis genes of Bax, caspase-3, and caspase-9. In conclusion, SFYXG could appear to attenuate myocardial injury by its antioxidative and antiapoptosis effect.

## 1. Introduction

Reactive oxygen species (ROS) is one of the leading causes of heart failure (HF) and cardiomyocytes' death. The generation and elimination of ROS, under normal circumstances, remain a balance in the process of oxidative metabolism. When under oxidative stress, the overproduction of ROS occurs and could transform the normal physiological signaling process into an abnormal one [1]. Recently, cardiomyocytes apoptosis, induced by ROS, has drawn increasing attention [2, 3]. Studies had shown that excessive ROS causes the disorder of energy metabolism and eventually leads to cellular apoptosis and necrosis, which is one of the pivotal causes of heart failure [4, 5]. Furthermore, H_2_O_2_, one classic type of ROS, acts as a damaging oxidant. Therefore, the best means to prevent heart failure are to eliminate the excessive production and accumulation of ROS, and to inhibit the apoptosis of myocardial cells. However, traditional Chinese medicine has been widely used in the comprehensive therapy of cardiovascular diseases, such as heart failure. Chinese medicines, with the effect of benefiting Qi and promoting blood circulation, could improve the heart function of rats with HF. SFYXG, a widespread used Chinese medicine prescription, has been demonstrated in the treatment of heart failure by its significant clinical effects. SFYXG is composed of eleven traditional Chinese medicines, including Renshen (Radix Ginseng), Fuzi (Radix Aconiti Carmichaeli), Guizhi (Ramulus Cinnamomi), Danshen (Radix Ginseng), Chishao (Radix Paeoniae Rubra), Yimucao (Herba Leonuri), Zhuling (Polyporus Umbellatus), Zexie (Rhizoma Alismatis), Tinglizi (Semen Descurainia), Sharen (Fructus Amomi), and Dazao (Fructus Jujubae). Its functions include nourishing Qi and warming Yang, promoting blood circulation, removing stasis, and promoting urination. Researches have shown that SFYXG could block renin-angiotensin system (RAS), improve the myocardial pathomorphology and ultrastructure, and inhibit the expression of c-fos and c-mycso in order to delay or improve cardiac remodeling in chronic HF rats. In addition, SFYXG could enhance the level of adenosine triphosphate (ATP) and improve cardiac in rats with HF by inhibiting the overexpression of uncoupling protein-2 (UCP-2) and weakening mitochondrion membrane potential. With the development of drug extraction technology, recent researches have indicated that GinsenosideRg5 [6], alkaloids [7], and tanshinone IIA [8, 9] have antioxidative properties, which are the main components of Renshen, Fuzi extract, and Danshen, respectively. Though both clinical and experimental studies have demonstrated that SFYXG has various pharmacological effects on heart failure, it remains unknown whether SFYXG could restrain H_2_O_2_-induced oxidative stress and apoptosis in myocardial cells.

## 2. Materials and Methods

### 2.1. Drug

SFYXG was provided by Shandong Buchang Pharmaceutical Co. Ltd. (lot no. 131101). 1g extract is equal to 9.5 g crude drug approximately. The drug was filtered by 0.22 *μ*m cell strainers in order to reach the required concentration by adding culture media.

### 2.2. Animals

Male/Female Sprague–Dawley rats (1–3 days old) were offered by Henan Laboratory Animal Center (license number: SCXK 2010–0002).

### 2.3. Isolation and Culture of Neonatal Rat Cardiomyocytes

The hearts of the rats were shredded into 1 mm [3] tissue fragments and digested 7-8 times. The equal amount of DMEM, containing 10% fetal bovine serum, was added to the supernatant solution, obtained through digestion, to make cell suspension. Cells were then collected through centrifugation at 1000 rpm for 10 min at 4°C, 3 times. Nonmyocytes were removed through 200 mesh screen. The majority of myocardial fibroblasts, after 90-minute cell differential adherent culture, were removed, and 0.1 mmol/L Brdu was added in order to inhibit the remnant myocardial fibroblasts growth. Following cells count, the myocardial cell suspension was diluted to required cell density and inoculated into 96-well plates (cell density 5 × 10^4^/mL, 100 *μ*L/hole) or 6-well plates (cell density 1 × 10^5^/mL, 2 mL/hole), and then incubated in CO_2_ incubator (37°C, 5% CO_2_). Culture media were changed every other day for 3–4 days. Experiments were carried out when cells pulsation rhythm synchronized and overlaid the bottom of the plates.

### 2.4. Effect of SFYXG, Coenzyme Q10, and H_2_O_2_ on Cardiomyocytes

Different concentrations of SFYXG (0, 0.1, 0.25, 0.5, 0.75, 1.0, and 1.5 mg/ml) were added to the 96-well plates, which were used to culture the primary cardiomyocytes. After incubation for 24 hours, 10 *μ*L MTT (5 mg/ml) was added to each pole and the incubation continued for 2 hours again. Then, the culture media were discarded and 100 *μ*L DMSO was added to each pole. The plates were oscillated for 10 minutes on the oscillator. Optical density (OD value) was measured with microplate reader at 490 nm.

Coenzyme Q10 at different concentrations (0, 1 × 10^−3^, 1 × 10^−4^, 1 × 10^−5^, 1 × 10^−6^, 1 × 10^−7^, and 1×10^−8^M) was added to the 96-well plates. Cell activity was determined by the same assay as shown above.

H_2_O_2_ at different concentrations (0, 10, 25, 50, 75, 100, and 150 *μ*M) was added to the 96-well plates, respectively, which were used to culture the primary cardiomyocytes to establish cell apoptosis model with the same method as stated above.

### 2.5. Effect of SFYXG on Oxidative Damage Induced by H_2_O_2_

The cardiomyocytes, cultured in the 96-well plates, were divided into 4 groups: the normal group, the model group, the SFYXG group, and the coenzyme Q10 group. The model, SFYXG, and coenzyme Q10 groups first dealt with the concentration of 50 *μ*M H_2_O_2_ for 6 h. Then, all the culture media were changed and the latter 2 groups were added, 0.5 mg/ml SFYXG and 1 × 10^−4^ M coenzyme Q10, respectively. After 24-hour incubation, OD value was detected by MTT.

In the following experiments, cell grouping and corresponding treatment were in accordance with the 4.5 method.

### 2.6. Calcein-AM Dyeing Method to Detect Cardiomyocytes Activity

Cells were treated as 4.5 method. 100 *μ*L calcein-AM (50 *μ*M) was added, and then cells were incubated for 25 min at 37°C, after being washed by PBS 3 times. Fluorescence microscope was used to observe cells after being washed by PBS 3 times. The fluorescence excitation wavelength is 490 nm, and the emission wavelength is 515 nm.

### 2.7. ROS Level in Cardiomyocytes

Cells were collected after corresponding treatment. Then, cells were incubated in DMEM medium with 50 *μ*L DCFH-DA (10uM) at 37°C for 30 min. Cells were washed with PBS 3 times, and the fluorescence intensity was measured.

### 2.8. AO/EB Staining

Cells were collected after the treatment and were adjusted to 1 × 10 ^10^/L suspension. Then, 5 *μ*L AO/EB equal volume mixture was added to the 95 *μ*L cell suspension. We took a drop of cell suspension and put it on the clean glass slide with a cover slip for observation in 3 min under the fluorescence microscope, whose excitation wavelength was 510 nm blue light.

### 2.9. JC-1 Assay to Detect the Mitochondrial Membrane Potential

Cells, which had been cultured and treated in 6-well plates afterwards, were harvested and 2 ml DMEM without serum was added and mixed in order to prepare cell suspension. Then, JC-1 detection solution was added to the mitochondrion suspension for chemical reaction in a dark environment for 7 min. According to the instructions, spectrofluorometer was implemented to detect the fluorescence intensity. The results were expressed as reflectivity [ER, ER = fluorescence intensity 590 nm/527 nm].

### 2.10. Transmission Electron Microscopy to Observe the Ultrastructure of Mitochondrion

After dealing with corresponding treatments, cells, cultured in the 6-well plates, were harvested. The 3% glutaraldehyde was added to centrifuge tube slowly in case of the cell cluster dispersed after being centrifuged at 1000 rpm for 10 min. They were fixed at 4°C for 2 hours.

### 2.11. The Apoptosis Rate of the Cardiomyocytes

The apoptosis rate of the cardiomyocytes was detected with the flow cytometer. The cardiomyocytes were washed by PBS 3 times and dissolved with trypsin. After that, the 1 × binding buffer was added to and diluted the cells to 5 × 10^6^ per milliliter and dyed by Annexin V (10 *μ*g/ml) and PI (10 *μ*g/ml) individually in the dark conditions for 3 min and 15 min, respectively. Lastly, the 1×binding buffer was replenished and it was made sure that the total volume was 500 *μ*l and detected through the flow cytometer.

### 2.12. The mRNA Expressions of Bcl-2, Bax, Caspase-3, Caspase-8, and Caspase-9

The mRNA expression levels in cardiomyocytes were quantitatively analyzed by rt-PCR. Under the ice bath, the total RNA of cardiac myocytes was extracted with Trizol reagent. The optical density ratio (A260/A280) of 260 nm and 280 nm (1.8–2.0) was measured by ultraviolet spectrophotometer. According to the instructions of reverse transcriptase kit, the reverse transcriptase reaction was carried out. Primer sequences are shown in Table 1

### 2.13. Protein Expressions of Bcl-2, Bax, Caspase-3, Caspase-8, and Caspase-9

The proteins were extracted by RIPA buffer with cocktail. Protein concentration was measured by the bicinchoninic acid assay (BCA assay). The total protein (5 *μ*g) was separated by SDS-PAGE and was transferred onto PVDF membranes. The membranes were blocked by 5% powdered milk in TBS-Tween 20 for 1 h at 25°C. The expression levels of proteins, related to apoptosis, were detected by using primary antibodies against Bcl-2 and Bax overnight at 4°C, and then combined with secondary antibody for 2 hours at 25°C. Blots, then, were developed by the Pierce ECL Plus Western Blotting substrate. ImageJ software was used to analyze the density. Cells were treated as 4.5 method. According to Caspase-Glo^®^3/8/9 Assay description, 100 *μ*L Caspase-Glo^®^3/8/9 reagent was added to the samples and washed with PBS 3 times; then, they would be mixed and incubated for 3 hours at 37°C. The luminescence was measured.

### 2.14. Statistical Analysis

All data are shown as mean ± SD. Statistical analysis was performed by one-way ANOVA when there are three or more groups. *P* < 0.05 is considered to indicate a statistically significant difference.

## 3. Results

### 3.1. Effect of SFYXG on the Activity of Neonatal Rat Cardiomyocytes

**Figure 1:**
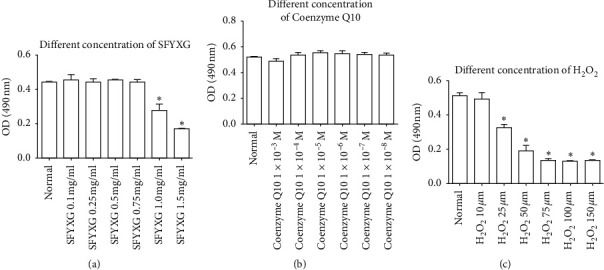
. Effect of SFYXG, coenzyme Q10, and H2O2 on the activity of neonatal rat cardiomyocytes. (a) Different concentration of SFYXG. (b) Different concentration of coenzyme Q10. (c) Different concentration of H_2_O_2_.

Based on the OD value, noncytotoxicity was found after SFYXG was applied within the range of concentration 0.1–0.75 mg/ml for 24-hour interaction, and when the concentration increased to 1.0 mg/ml and more, cell activity decreased significantly compared with the normal group (*P*^*∗*^<0.05; Figure 1(a)). Therefore, in the follow-up experiments, 0.5 mg/ml SFYXG was chosen as the intervention concentration. Coenzyme Q10 1 × 10^–4^ M and H_2_O_2_ 50 *μ*M were chosen by using the same method (Figures 1(b) and 1(c)).

### 3.2. Effect of SFYXG on Oxidative Stress Injury

**Figure 2:**
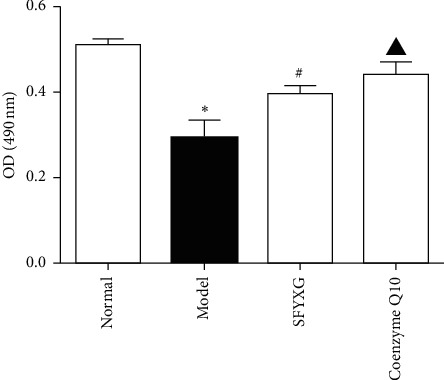
The activity of cardiomyocytes was detected by MTT. Note: *P*^*∗*^<0.05 vs normal group (0.29 ± 0.04 vs 0.51 ± 0.02); *P*^#^<0.05 vs model group (0.40 ± 0.02 vs 0.29 ± 0.0 4); *P*^▲^<0.05 vs model group (0.44 ± 0.03 vs 0.29 ± 0.0 4).

**Figure 3:**
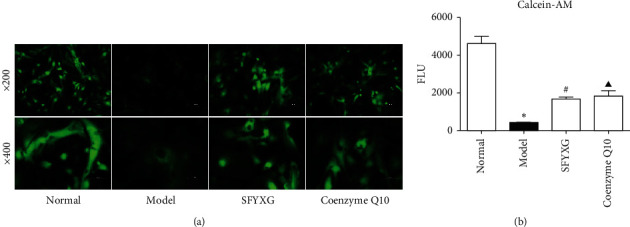
The activity of cardiomyocytes was determined by calcein-AM method. *P*^*∗*^<0.05 vs normal group (392.70 ± 32.84 vs 4626.11 ± 383.38); *P*^#^<0.05 vs model group (1645.30 ± 112.35 vs 392.70 ± 32.84); *P*^▲^<0.05 vs model group (1808.44 ± 289.77 vs 392.70 ± 32.84). The fluorescence excitation wavelength is 490 nm and the emission wavelength 515 nm.

Compared with normal group, the activity of cardiomyocytes in the model group significantly decreased (*P* < 0.05; Figures 2 and 3). 0.5 mg/ml SFYXG and coenzyme Q10 1 × 10^−4^ M both promoted activity of cardiomyocytes (*P* < 0.05; Figures 2 and 3).

### 3.3. Effect of SFYXG on ROS Level

**Figure 4:**
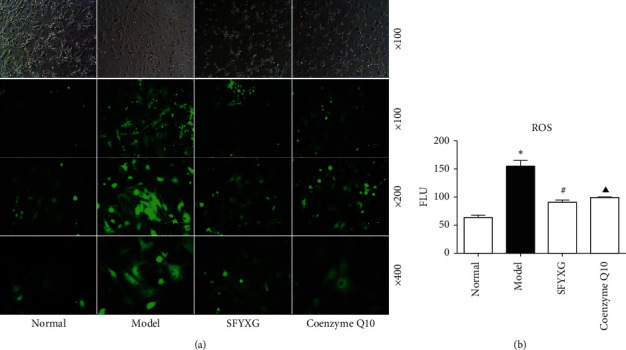
Effect of SFYXG on ROS level. Note: (a) the green fluorescence intensity is proportional to the ROS level. The fluorescence excitation wavelength is 488 nm and the emission wavelength 525 nm. ROS level was tested by microplate reader (b).

Compared with the normal group (Figure 4), ROS level increased (154.82 ± 10.01 vs. 63.88 ± 3.75，*P*^*∗*^<0.05) after 50 *μ*M H2O2 was applied for 6 hours, whereas ROS levels (89.18 ± 3.45 vs 154.82 ± 10.01, 98.82 ± 1.27; *P*^#^<0.05, *P* < 0.05^▲^; Figure 4) decreased after 0.5 Mg/Ml SFYXG and coenzyme Q10 1 × 10^−4^ M were applied, respectively. The results suggested that SFYXG could weaken ROS.

### 3.4. Effect of SFYXG on Cardiomyocytes Morphology

**Figure 5:**
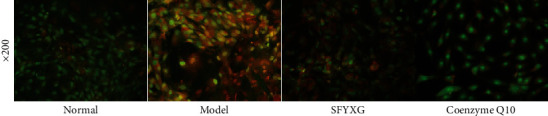
SFYXG influence on cardiomyocytes morphology.

Under fluorescence microscope, cells in the normal group were full with intact membrane, and the nuclei appeared homogeneous green fluorescence. However, the shape of cells in the model group was irregular, and the apoptotic cells showed inhomogeneous orange red fluorescence. Compared with the model group, the majority of the cells in SFYXG and coenzyme Q10 groups presented green fluorescence and the orange red fluorescence decreased (Figure 5). The results show that SFYXG attenuates cardiomyocytes' morphology injury caused by H2O2.

### 3.5. SFYXG Influence on Cardiomyocytes MMP

**Figure 6:**
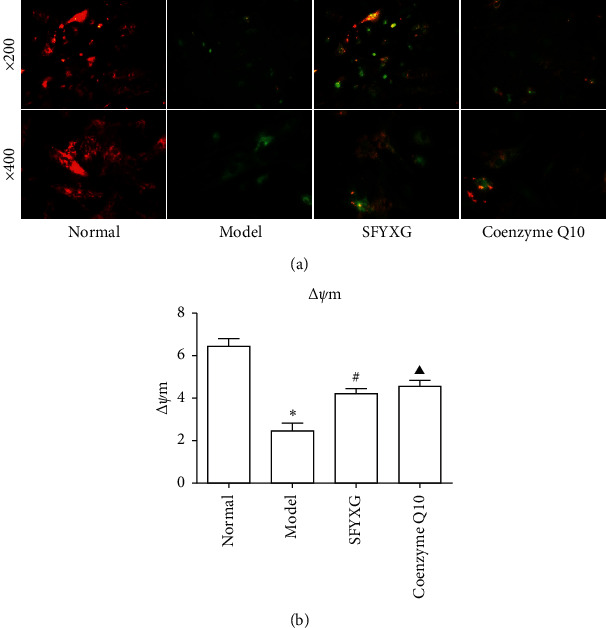
SFYXG influence on cardiomyocytes MMP. Note: mitochondrion membrane potential (MMP) changes were observed by fluorescence microscope (a) and the MMP level was tested by microplate reader (b).

Compared with the normal group, cardiomyocytes MMP caused by H2O2 decreased rapidly in the model group (2.44 ± 0.32 vs 6.45 ± 0.35, *P*^*∗*^<0.05). What is more, in SFYXG and coenzyme Q10 groups, the MMP were enhanced significantly (4.19 ± 0.24, 4.55 ± 0.26; *P*^#^ <0.05, *P*^▲^ <0.05; Figure 6). It suggested that SFYXG could reduce the toxicity of H2O2.

### 3.6. Ultrastructure Changes of Mitochondrion in Each Group

**Figure 7:**
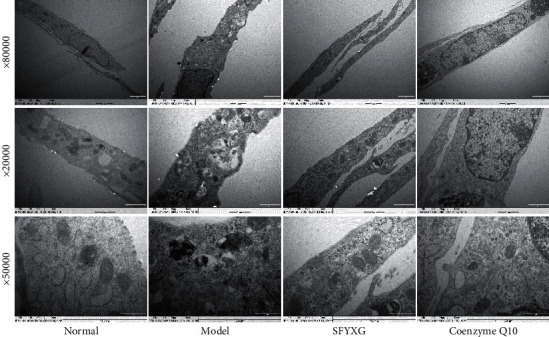
SFYXG effect on mitochondrion ultrastructure.

In the normal group, the mitochondria of individuals are homogeneous with integrate structure, and mitochondrion cristae are clear and complete. And the myocardial fibers are aligned. However, in the model group, the mitochondria are in various sizes and shapes, and they swell irregularly with incomplete structure. Above this, mitochondria dissolved into the vacuole, and partial creast and myocardial fibers fracture are broken and fuzzy. Compared with the model group, the SFYXG group mitochondria swell slightly, the cristae and myocardial fibers are in a regular pattern, and there is slight dissolution (Figure 7).

### 3.7. Apoptosis Rate of Primary Cardiomyocytes In Vitro of Each Group

**Figure 8:**
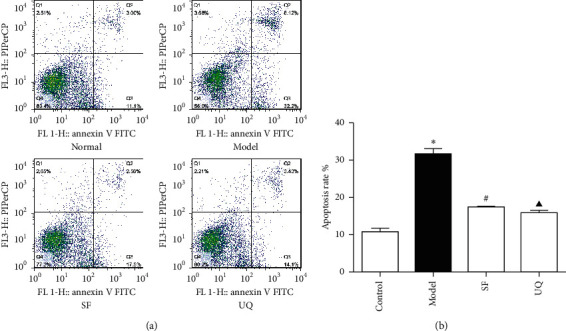
Effect of SFYXG on apoptosis rate. Note: apoptosis rate was detected by the flow cytometer (a) and the data were analyzed by FlowJo7.6 (b).

Compared to the normal group, the apoptosis rate was higher than the model group (10.73 ± 0.46 vs 31.46 ± 0.78, *P*^*∗*^<0.05). What is more, in SFYXG and coenzyme Q10 groups, the apoptosis rate was reduced significantly (17.26 ± 0.31, 15.67 ± 0.43; *P*^#^ <0.05, *P*^▲^ <0.05; Figure 8). It suggested that SFYXG could reduce the apoptosis rate of the cardiomyocytes caused by H2O2 (Figure 8).

### 3.8. Effect of SFYXG on mRNA Expressions of Bax, Caspase-3, Caspase-8, Caspase-9, and Bcl-2

**Figure 9:**
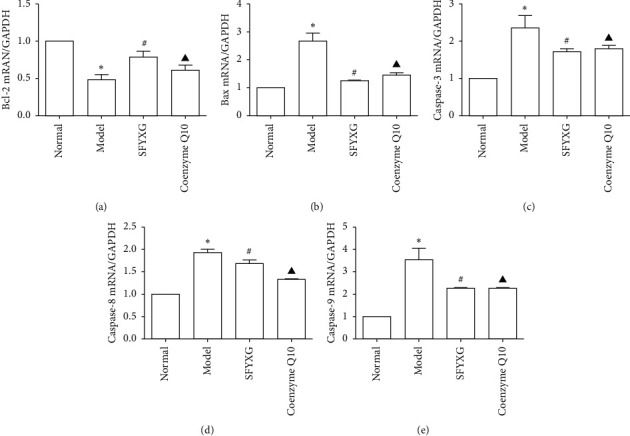
Effect of SFYXG on mRNA expressions of the corresponding genes. Note: *P*^*∗*^<0.05 was compared with the normal group; *P*^#^<0.05 and *P*^▲^<0.05 were compared with the model group.

Compared with the normal group, cardiomyocytes showed higher expression levels of Bax, caspase-3, caspase-8, and caspase-9, and lower expression level of Bcl-2 mRNA in the apoptosis model. After treatment with SFYXG and coenzyme Q10, Bax, caspase-3, caspase-8, and caspase-9 in cardiomyocytes decreased significantly, whereas Bcl-2 mRNA expression increased (*P* < 0.05; Figure 9).

### 3.9. Effect of SFYXG on Protein Expressions of Bax, Bcl-2, Caspase-3, Caspase-8, and Caspase-9

**Figure 10:**
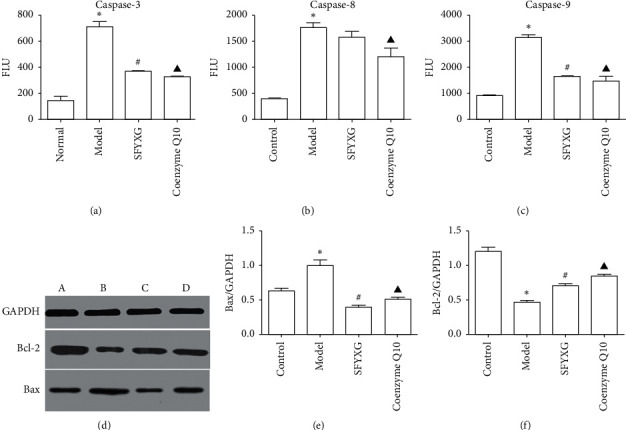
Effect of SFYXG on protein expressions of the corresponding factors. Note: caspase-3, caspase-8, and caspase-9 were detected by Caspase-Glo®3 Assay, Caspase-Glo®8 Assay, and Caspase-Glo®9 Assay, respectively; Bcl-2 and Bax were detected by Western blot. *P*^*∗*^<0.05 was compared with the normal group; *P*^#^<0.05 and *P*^▲^<0.05 were compared with the model group.

The results show that the proapoptosis proteins expressions of Bax, caspase-3, caspase-8, and caspase-9 notably increased, while the expression of the antiapoptosis Bcl-2 decreased in the apoptosis model. The expression levels of the related proteins were significantly reversed by SFYXG (*P* < 0.05; Figure 10), which demonstrates that SFYXG could regulate cardiomyocytes apoptosis through Bax, caspase-3, caspase-9, and Bcl-2. From the results, we also conclude that SFYXG decreased caspase-8 expression, but there were no statistical differences.

## 4. Discussion

In fact, “heart failure” has been found in traditional Chinese medicine literature. However, it refers to the deficiency of heart-qi and heart-blood, which is quite different from the “heart failure” in the western medicine. Based on the clinical characteristics, heart failure can be included in the diseases of “asthma,” “palpitation,” and “edema,” which are now unanimously named “heart failure disease” (HFD). In the late stage of HFD, the insufficiency of heart-qi and heart-yang leads to blood stasis in the heart. SFYXG is usually used to benefit heart-qi, warm heart-yang, and promote blood circulation in order to remove blood stasis. Previous studies have demonstrated that SFYXG could downregulate Ang II and block the RASS system, so as to improve myocardial pathological morphology and myocardial ultrastructure to delay myocardial remodeling. The present research observed that SFYXG could improve the apoptotic myocardiocytes caused by H2O2 through activating myocardial cells under oxidative stress, and weakening the excessive ROS. Then, the apoptosis rate decreases. In addition, SFYXG reduces the number of apoptotic cardiomyocytes and improves the cell morphology. Furthermore, it is found that SFYXG could improve the morphology of mitochondrion, protect the integrity of mitochondrion structure, reduce vacuolar change, and decrease the dissolution of mitochondrion crista and myocardial fibers.

As is well known, mitochondrion is where life activities take place, including cell respiratory chain, oxidative phosphorylation, and cell apoptosis. The electron transfer chain of the mitochondrion is the main place for the production of ROS [10, 11]. Normally, a small amount of ROS produced in mitochondrion is essential for the cell functions, including the defense, detoxification, and synthesis of some important substances. However, when HF occurs, the activity of antioxidant enzymes such as superoxide dismutase (SOD) decreases, and the scavenging of ROS declines, which result in the excessive accumulation of ROS. However, the excessive ROS damages the mitochondrion, decreases the MMP, changes the permeability of mitochondrion membrane, and induces cardiomyocyte apoptosis through mitochondrion signal transduction pathway [12–14].

It is noteworthy that the Bcl-2 family and the caspase family are closely related to cardiomyocyte apoptosis [15, 16]. The Bcl-2 family contains more than 20 homologous proteins, which can be divided into anti-apoptotic protein and proapoptotic protein. The typical antiapoptotic members, such as Bcl-2, Bcl-xL, Mcl-1, and Bcl-W, are mainly found in the mitochondrion outer membrane. The major proapoptotic protein, Bax, for example, is homologous to the amino acid sequence of Bcl-2. And Bax exists in the cytoplasm in the form of monomer in the normal cells. When the cells are stimulated by the associated apoptotic signal, they will be transferred to the mitochondrion to change the MMP and mitochondrion permeability, and to promote the release of cytochrome c (apoptotic active substance). Additionally, Bcl-2 is found to interact with Bax to form heterodimer, and thus prevent Bax release and inhibit cell apoptosis [17–19]. This study examines the changes in MMP, the mRNA, and protein expression of Bcl-2 and Bax under the cardiomyocytes under oxidative stress induced by H2O2. The results show that the MMP of the cells in the model group deceases significantly, whereas there is recovery after treatment by SFYXG. In the model group, the mRNA and protein expression level of Bcl-2 decreases and Bax increases. The results present that the changes are both corrected by SFYXG. The results suggest that the effect of SFYXG on apoptotic cardiomyocyte induced by H_2_O_2_ could be attributed to its ability to recover the stability of MMP and the expression of Bax and Bcl-2.

Caspase belongs to cysteine protease. Once activated by the signal transduction pathway, caspase could degrade the protein and cause irreversible cell death. The caspase family, involved in cell apoptosis, is divided into two categories: one is the executioner, such as caspase-3, which could degrade the structural and functional protein and lead to direct apoptosis; the other is the initiator, such as caspase-8 and caspase-9, which could be activated by self-shearing and causes the cascade reaction. It is generally believed that caspase-8 mediates apoptosis in the death receptor pathway [20]. Caspase-3 is activated accordingly followed by the activating caspase-9, and they are both involved in mitochondrion apoptosis pathway [21, 22]. Overexpression of Bcl-2 on mitochondrion membrane could avoid cell apoptosis by inhibiting the changes of mitochondrion permeability, reducing the release of cytochrome C, and inhibiting caspase activation. Furthermore, overexpression of Bcl-2 causes the accumulation of glutathione (GSH) in the nucleus, the change of redox equilibrium, and weakens caspase activity [23]. When cells are subjected to oxidative stress, ROS and caspase interact with each other to promote cell apoptosis. Our results show that the expression of caspase-9 and caspase-3 increases significantly after the treatment of H_2_O_2_, but decreases significantly after the treatment of SFYXG, which indicates that oxidative stress could activate not only the Bcl-2 in the upstream of the mitochondrion, but also caspase. However, no remarkable change is found in caspase-8, which may be related to intermediation of the death receptor pathway.

## 5. Conclusion

SFYXG could inhibit cardiomyocytes apoptosis. It may raise cell viability by clearing the excessive ROS, protecting the morphology and structure of mitochondrion, stabilizing MMP, downregulating mRNA and protein expression of Bax, caspase-3, and caspase-9, and upregulating Bcl-2. This study may provide a theoretical and experimental basis for the clinical application of SFYXG in the prevention and treatment of heart failure.

## Figures and Tables

**Table 1:** The Primer sequences of RT-PCR.

Gene	Sense primer (F)	Reverse primer (R)	Fragment length (bp)
GAPDH	ACA GCA ACA GGG TGG TGG AC	TGAG GGT GCA GCG AACTT	252
Bcl-2	CTT TGA GTT CGG TGG GGT CA	AGT TCC ACA AAG GCA TCC CAG	153
Bax	GCT CAA GGC CCT GTG CAC TAA	GAA GCC TCA GCC CAT CTT CTT	223
Caspase-3	GAG CTG GAC TGC GGT ATT GA	AGG AAT AGT AAC CGG GTG CG	118
Caspase-8	GAC CAC ATC CCG CAG AAG AA	GAT CCC GCC GAC TGA TAT GG	139
Caspase-9	CAT CTT CAA TGG GAC CGG CT	GGT CTT TCT GCT CAC CAC CA	86

## Data Availability

All data were acquired from the Center Lab of the First Affiliated Hospital of Henan University of CM.
